# Expanding role of absolute zero fluoroscopy atrial septal defect closure: a single-center experience

**DOI:** 10.3389/fcvm.2025.1430555

**Published:** 2025-04-04

**Authors:** Radityo Prakoso, Rina Ariani, Yovi Kurniawati, Sisca Natalia Siagian, Aditya Agita Sembiring, Damba Dwisepto Aulia Sakti, B. R. M. Ario Soeryo Kuncoro, Brian Mendel, Estu Rudiktyo, Amiliana Mardiani Soesanto, Olfi Lelya, Oktavia Lilyasari

**Affiliations:** ^1^ Division of Pediatric Cardiology and Congenital Heart Disease, Department of Cardiology and Vascular Medicine, National Cardiovascular Center Harapan Kita, Universitas Indonesia, Jakarta, Indonesia; ^2^ Division of Non-invasive Diagnostic and Cardiovascular Imaging, Department of Cardiology and Vascular Medicine, National Cardiovascular Center Harapan Kita, Universitas Indonesia, Jakarta, Indonesia; ^3^ Department of Cardiology and Vascular Medicine, National Cardiovascular Center Harapan Kita, Universitas Indonesia, Jakarta, Indonesia

**Keywords:** atrial septal defect, percutaneous, transesophageal echocardiography, zero-fluoroscopy, fluoroscopy

## Abstract

**Introduction:**

Zero-fluoroscopy, exclusively ultrasound-guided atrial septal defect (ASD) catheter closure has been reported. However, data on the effectiveness of this technique in complex cases remains limited.

**Objectives:**

This study aims to evaluate the safety, efficacy, and outcomes of ASD catheter closure using exclusive ultrasound guidance, with a particular focus on complex cases.

**Methods:**

We conducted a retrospective review of clinical data from patients who underwent attempted ASD catheter closure with exclusive ultrasound guidance at our institution between July 2018 and April 2024. Patients were categorized into two groups based on the complexity of their cases (simple vs. complex ASD cases). Complex cases included patients with large defects (≥25 mm), multiple or fenestrated ASDs, deficient posterior-inferior rim <3 mm, deficient retro-aortic rim <5 mm, pulmonary hypertension, septal malalignment, and pregnancy. We analyzed and compared demographic information, procedural data, and outcomes between the two groups.

**Results:**

We identified 339 patients (18.2% males, 53.6% adults) with a median age of 21 years (IQR, 9–38) and median weight of 46.5 Kg (IQR, 22–59). Overall, median defect size was 20 mm (IQR, 16–25) and device size was 26 mm (IQR, 20–32). 248 (73.1%) patients were classified as complex including 98 (28.9%) with large defects (≥25 mm), 33 (9.7%) with multiple or fenestrated ASDs, 53 (15.6%) with pulmonary hypertension, 171 (50.4%) with rim deficiency, 50 (14.7%) with septal malalignment, and 6 (1.7%) with pregnancy. Two procedures (0.5%) were guided using transthoracic ultrasound and 337 (99.4%) using both transthoracic and transoesophageal ultrasound. The implantation success rate was 98.9% in simple cases and 97.1% in complex cases (*p* < 0.001). The rate of conversion to fluoroscopy guidance was 0 (0%) in simple cases and 7 (2.8%) in complex cases (*p* < 0.001). The median procedural time was 41 min (IQR, 30–47) in simple cases and 45 min (IQR, 36–62) in complex cases (*p* = 0.008). Sixteen patients (4.7%) underwent balloon-assisted procedures, and 12 (3.5%) required redeployment. There were 6 (1.7%) serious procedural complications (0 in simple cases, 6 in complex cases). The median follow-up was 187 days (IQR, 21–428.7). There were no residual shunt at latest follow-up for both simple and complex cases.

**Conclusions:**

Zero-fluoroscopy exclusively echocardiography-guided ASD closure is effective in both simple and complex cases. However, the rate of conversion to fluoroscopy and implantation failure are significantly higher in complex ASD cases.

## 1 Introduction

Atrial septal defect (ASD) is one of the most frequently encountered congenital heart diseases, with an estimated prevalence of 3.89 per 1,000 children and 0.88 per 1,000 adults (1). The clinical course of ASD is variable and depends on the lesion characteristics. Early diagnosis and treatment of ASD can avoid serious complications. Management of ASD involves percutaneous device closure or open-heart surgery (2–4). Percutaneous closure of ASD under fluoroscopic guidance is now considered a routine procedure and studies using a variety of devices have reported good success with low complication rates in children and adults (5).

Nevertheless, radiation exposure during fluoroscopy represents stochastic and deterministic effect to the patient, especially for small infants and pregnant woman (6–8). Because percutaneous ASD closure under fluoroscopic guidance is usually carried out with the assistance of transesophageal echocardiography (TEE) or transthoracic echocardiography (TTE), it has been suggested that the exclusive use of echocardiography to guide ASD closure could be used to guide device placement (9). Many studies have reported the use of echocardiography-guided only to guide ASD closure without fluoroscopy (10–15). Nevertheless, data about the efficacy and safety of exclusive echocardiography-guidance in complex ASD closures, such as large defects, multiple or fenestrated ASDs, deficient rims, pulmonary hypertension, septal malalignment, and pregnancy, are limited. Therefore, the aim of this retrospective study was to evaluate comprehensively our experience of exclusive use of echocardiography to guide ASD closure in complex conditions in our hospital.

## 2 Methods

### 2.1 Study population and design

This retrospective study included patients who underwent percutaneous closure of ASD at our institution from July 2018 to April 2024. Patients were categorized into two groups based on the complexity of their cases (simple vs. complex ASD cases). Complex cases included patients with large defects (≥25 mm), multiple or fenestrated ASDs, deficient posterior-inferior rim <3 mm (The posterior aspect of the postero-inferior rim is located toward the back of the heart, near the entry of the inferior vena cava/IVC, while the inferior aspect lies below the defect, closer to the tricuspid valve and coronary sinus), deficient retro-aortic rim <5 mm, pulmonary hypertension, septal malalignment, and pregnancy. We analyzed and compared demographic information, procedural data, and outcomes between the two groups. The closure procedure was conducted using transthoracic echocardiography and/or transesophageal echocardiography guidance exclusively, without the use of fluoroscopy. Inclusion criteria comprised a preoperative diagnosis of ASD, established through medical history, clinical manifestations, electrocardiogram findings, and transthoracic echocardiography (TTE), and deemed suitable for percutaneous closure following an outpatient TTE assessment from standard subcostal, apical 4-chamber, and parasternal short-axis views. These criteria included: (1) ASD with a diameter between ≥5 mm and ≤40 mm, contributing to increased right ventricular volume load; (2) distances from the defect edge to the superior vena cava, coronary sinus, and pulmonary vein ≥5 mm, and from the defect edge to the atrioventricular valve ≥7 mm; and (3) atrial septum diameter larger than the diameter of the left atrial side of the chosen occluder. The study received approval from the institutional ethics committee of the National Cardiovascular Center Harapan Kita, and informed written consent was obtained from patients or their legal guardians before the procedure.

### 2.2 Study variables

Baseline demographic and clinical characteristics, including gender, body weight, age, size of the defect, and presence of deficient rims were documented. Additionally, valve abnormalities, arrhythmia types, any additional diagnoses, history of previous intervention/surgery, and procedural specifics were extracted from medical records.

### 2.3 Procedure

**Figure 1:**
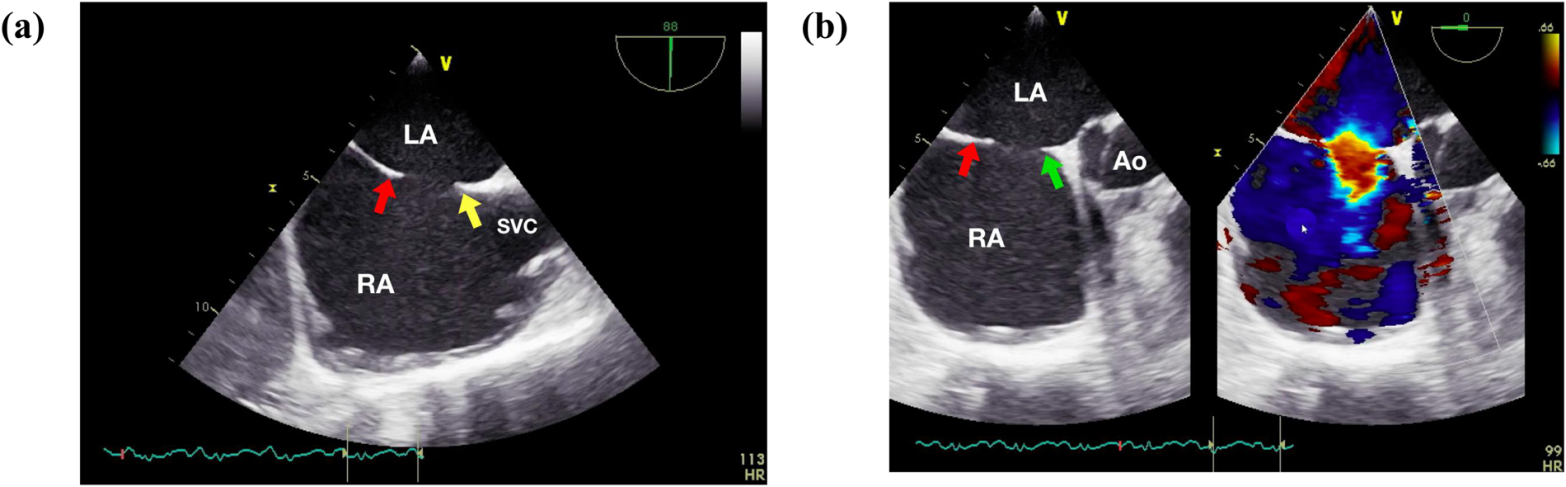
Initial assessment of the secundum atrial septal defect (ASD). **(a)** Initial assessment involves evaluating the SVC and IVC rim at 80°–100°. **(b)** Subsequently, the posterior, mitral, and inferior rims are assessed at 0°. Ao, aorta; LA, left atrium; RA, right atrium; SVC, superior vena cava. Red arrow showed postero-inferior rim, yellow arrow showed superior rim, green arrow showed aortic rim.

The majority of procedures were performed by visiting operators. Five main operators, all of whom were well-acquainted with the standardized technique for fluoroscopy-guided atrial septal defect (ASD) closure, performed the procedures. All patients underwent general anesthesia with endotracheal intubation. Following intubation, comprehensive studies utilizing TTE and/or TEE were conducted to assess various aspects of the ASD anatomy, including location, size, presence of additional defects, and adequacy of different rims (see Figures 1a,b). The defect size was determined based on its maximum diameter. Balloon sizing is traditionally considered an essential step in the transcatheter closure of secundum ASD, as balloon inflation alters the defect's shape to approximate the balloon's circular form. However, based on our experience, percutaneous closure can often be achieved without balloon sizing, provided the defect's size and morphology are accurately assessed, except in cases of particularly large defects. Subsequently, an occluder was chosen for each patient based on the TTE or TEE findings, with a diameter exceeding the maximum defect diameter by 2–4 mm. Vascular access was obtained via the right femoral vein. In patients with a mean pulmonary artery (PA) pressure exceeding 25 mmHg, an oxygen vasoreactivity test was performed. If the resulting pulmonary vascular resistance (PVR) to systemic vascular resistance (SVR) ratio was less than 0.33, transcatheter closure of the ASD was deemed appropriate.

**Figure 2:**
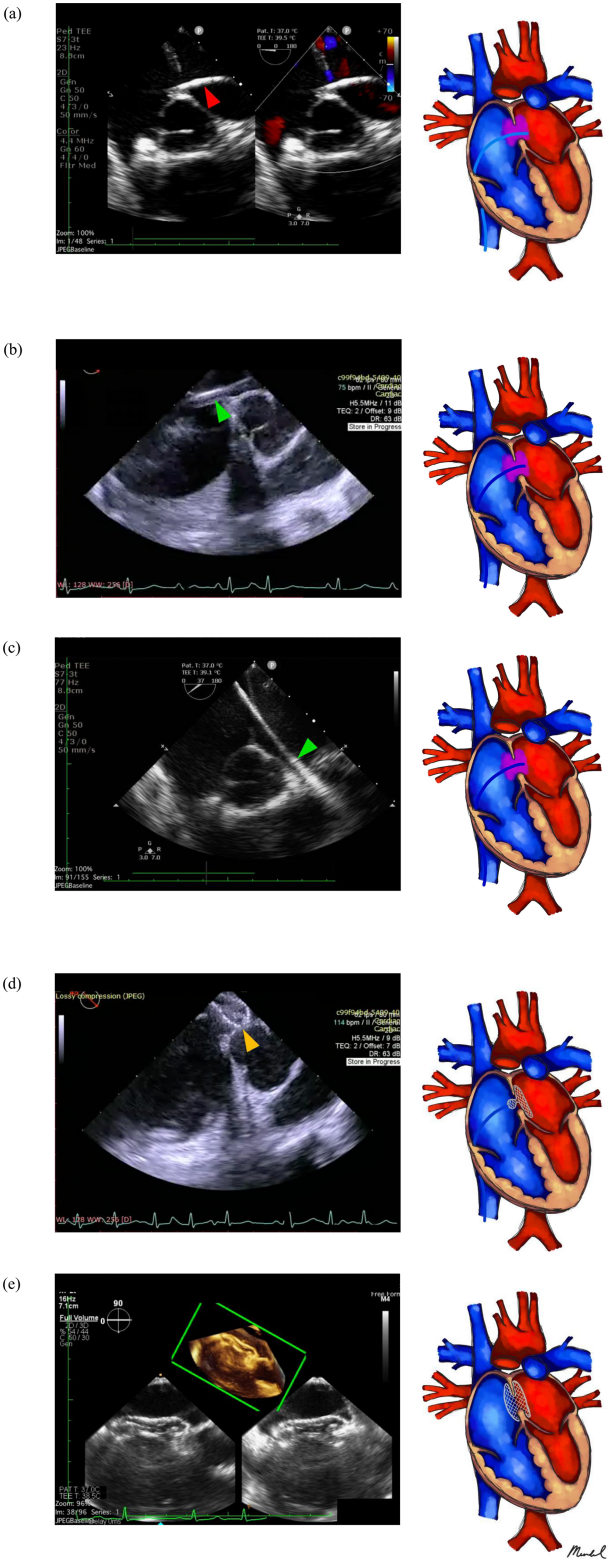
Standardized percutaneous zero fluoroscopy ASD closure procedure implemented in our center. **(a)** Atrial septum crossing is performed using TEE, ME-SAX, and/or ME bicaval view. Following crossing, the catheter (red arrowhead) is positioned in the left-sided PV preferably, and an exchange wire position is securely established in the left (usually upper) PV before introducing the delivery sheath (green arrowhead). **(b,c)** The delivery sheath is inserted in the ME-SAX view. **(d)** Device deployment follows (orange arrowhead). **(e)** Lastly, device evaluation before and after detachment is conducted.

Heparin was administered at a dosage of 100 IU/kg along with antibiotic prophylaxis. A 0.035-inch J-tipped super-stiff Amplatzer^TM^ guidewire (AGA Medical Corp, Golden Valley, MN) was guided into the right atrium (RA) under TEE using bicaval and midesophageal short-axis views. Subsequently, a MPA2 diagnostic catheter (Cordis, Johnson & Johnson, Warren, NJ, USA) was advanced into the RA along the wire. The guidewire was then gradually withdrawn while the catheter tip was monitored by TEE. Clockwise torque on the catheter might be necessary to ensure proper direction as it traversed the defect towards the left atrium and the pulmonary vein (see Figure 2a).

Under catheter guidance, the guidewire was advanced through the defect and into the pulmonary vein, after which the catheter was removed (Figure 2a). The delivery sheath and dilator were then advanced into the femoral vein over the guidewire, with TEE monitoring the inferior vena cava using bicaval view. Advancing the sheath over the wire without the dilator into a pulmonary vein is a potentially dangerous step, as it may lead to perforation. Upon detection of the sheath and dilator by TEE, the dilator was retracted into the sheath to visualize the sheath tip clearly. Tracked by TEE, the sheath was subsequently advanced to the pulmonary vein (see Figures 2b,c).

An appropriately sized occluder was introduced into the delivery sheath until reaching the sheath tip. With TEE guidance, the sheath was gently withdrawn to deploy the left disc and waist in the left atrium, then retracted until the disc was against the atrial septum. The right side of the device was deployed by retracting the delivery sheath while applying slight cable tension (see Figure 2d). Prior to unscrewing the device, its correct and stable position was confirmed by TEE, ensuring unobstructed flow from the coronary sinus, pulmonary veins, superior and inferior vena cavae, and competence of the atrioventricular valves (see Figure 2e).

### 2.4 Post-operative care and follow-up

Following the procedure, all patients were prescribed daily aspirin (5 mg/kg) for six months. Clinical examination, electrocardiography, and transthoracic echocardiography (TTE) were conducted at various intervals: 1 day post-procedure, before discharge, 1 month, 6 months, and 1 year after discharge, and annually thereafter. Procedural success was defined by specific criteria: a well-positioned device occluder as confirmed by TTE at 24 and 72 h post-procedure, absence of device occluder migration, no need for conversion to fluoroscopy during the procedure, and discharge from the hospital by post-procedure day 3.

### 2.5 Statistical analysis

Categorical data were summarized as counts (*n*) and percentages (%), while numerical data were reported as medians with interquartile ranges (IQR). For comparing categorical variables, Chi-square or Fisher's exact tests were applied, and the independent Mann–Whitney test was used for analyzing numerical data. Associations between outcomes were assessed using Spearman's correlation coefficients. All statistical analyses were conducted using SPSS version 26.0, with a significance threshold of *p* < 0.05.

## 3 Results

### 3.1 Baseline characteristics

We identified 339 patients (18.2% males, 53.6% adults) with a median age of 21 years (IQR, 9–38) and median weight of 46.5 Kg (IQR, 22–59). Overall, median defect size was 20 mm (IQR, 16–25). 248 (73.1%) patients were classified as complex including 98 (28.9%) with large defects (≥25 mm), 33 (9.7%) with multiple or fenestrated ASDs, 53 (15.6%) with pulmonary hypertension, 171 (50.4%) with rim deficiency, 50 (14.7%) with septal malalignment, and 6 (1.7%) with pregnancy (see Table 1).

**Table 1:** Baseline demographic and clinical characteristics.

	*N* (overall %)	Simple cases (*N* = 91)	Complex cases (*N* = 248)	*P* value
Male, *n* (%)	62 (18.2)	20 (21.9)	42 (16.9)	0.178
Weight, kg, median (IQR)	46.5 (22–59)	40 (17–54)	49 (27–60)	0.094
Age, years, median (IQR)	21 (9–38)	15 (6.5–32.7)	23 (11–40)	0.090
Defect size, mm, median (IQR)	20 (16–25)	17 (13–20)	22 (18–27)	0.050
Large defect (≥25 mm), *n* (%)	99 (29.2)	0 (0)	99 (39.9)	
Multiple or fenestrated ASD, *n* (%)	31 (9.1)	0 (0)	31 (12.5)	
Deficient posterior-inferior rim <3 mm, *n* (%)	53 (15.6)	0 (0)	53 (21.3)	
Deficient retro-aortic rim <5 mm, *n* (%)	162 (47.7)	0 (0)	162 (65.3)	
Pulmonary hypertension, *n* (%)	114 (33.6)	0 (0)	114 (45.9)	
Septal malalignment, *n* (%)	23 (6.7)	0 (0)	23 (9.2)	
Pregnancy, *n* (%)	7 (2)	0 (0)	7 (2.8)	

ASD, atrial septal defect; AV, atrioventricular; BPV, balloon pulmonary valvuloplasty; IQR, interquartile range; MVR, mitral valve replacement; TVR, tricuspid valve repair; VSD, ventricular septal defect; VT, ventricular tachycardia.

### 3.2 Procedural details

Device size was 26 mm (IQR, 20–32). The types of devices used included Amplatzer^TM^ Septal Occluder (AGA Medical Corp, Golden Valley, MN) [16 (4.7%)], Figulla Flex II ASD Occluder (Occlutech, Germany) [2 (0.5%)], Memopart^TM^ ASD Occluder (Lepu, Beijing) [61 (17.9%)], Ceraflex^TM^ ASD Occluder (Lifetech Scientific, Shenzhen) [15 (4.4%)], and Cera^TM^ ASD Occluder (Lifetech Scientific, Shenzhen) [245 (72.2%)]. Two procedures (0.5%) were guided using transthoracic ultrasound and 337 (99.4%) using both transthoracic and transoesophageal ultrasound. The implantation success rate was 98.9% in simple cases and 97.1% in complex cases (*p* < 0.001). The rate of conversion to fluoroscopy guidance was 0 (0%) in simple cases and 7 (2.8%) in complex cases (*p* < 0.001). The median procedural time was 41 min (IQR, 30–47) for simple cases and 45 min (IQR, 36–62) for complex cases (*p* = 0.008). Sixteen patients (4.7%) underwent balloon-assisted procedures, and 12 (3.5%) required redeployment. The median follow-up was 187 days (IQR, 21–428.7) (see Table 2). Balloon-assisted techniques were employed in five patients to address large defects, malalignment, or device protrusion. Seven serious adverse events (SAEs) occurred in 7 patients all of which had complex ASDs. The characteristics of these seven patients are outlined in Table 3.

**Table 2:** Procedural details of zero fluoroscopy atrial septal defect (ASD) closure.

	*N* (overall %)	Simple cases (*N* = 91)	Complex cases (*N* = 248)	*P* value
Guidewire position				
Left atrium, *n* (%)	29 (85.5)	6 (6.5)	23 (9.2)	
Left upper pulmonary vein, *n* (%)	274 (80.8)	74 (81.3)	200 (80.6)	
Right upper pulmonary vein, *n* (%)	35 (10.3)	11 (12)	24 (9.6)	
Right lower pulmonary vein, *n* (%)	1 (0.2)	0 (0)	1 (0.4)	
Deployment				
Left atrium, *n* (%)	304 (89.6)	80 (87.9)	224 (90.3)	
Left upper pulmonary vein, *n* (%)	33 (9.7)	11 (12)	22 (8.8)	
Right upper pulmonary vein, *n* (%)	2 (0.5)	0 (0)	2 (0.8)	
Convert to fluoroscopy (*n*, %)	7 (2)	0 (0)	7 (2.8)	<0.001
Device size, mm, median (IQR)	26 (20–32)	20 (16–24)	28 (22–32)	0.006
Device types				
Amplatzer^TM^ Septal Occluder (AGA Medical Corp, Golden Valley, MN), *n* (%)	16 (4.7)	5 (5.4)	11 (4.4)	
Figulla Flex II ASD Occluder (Occlutech, Germany), *n* (%)	2 (0.5)	0 (0)	2 (0.8)	
Memopart^TM^ ASD Occluder (Lepu, Beijing), n(%)	61 (17.9)	17 (18.6)	44 (17.7)	
Ceraflex^TM^ ASD Occluder (Lifetech Scientific, Shenzhen), *n* (%)	15 (4.4)	7 (7.6)	8 (3.2)	
Cera^TM^ ASD Occluder (Lifetech Scientific, Shenzhen), *n* (%)	245 (72.2)	62 (68.1)	183 (73.7)	
Procedural time, mins, median (IQR)	40.5 (31–55.7)	41 (30–47)	45 (36–62)	0.008
Balloon-assisted, *n* (%)	23 (6.7)	0 (0)	23 (9.2)	<0.001
Redeployment, *n* (%)	7 (2)	2 (2.1)	5 (2)	<0.001
Complications	27 (7.9)	6 (6.5)	21 (8.4)	<0.001
Intraprocedural, *n* (%)	18 (5.3)	4 (4.3)	14 (5.6)	<0.001
Postprocedural, *n* (%)	9 (2.6)	2 (2.1)	7 (2.8)	<0.001
Residual shunt, *n* (%)	18 (5.3)	3 (3.2)	15 (6)	<0.001
Death, *n* (%)	0 (0)	0 (0)	0 (0)	
Success rate, *n*/*N* (%)	331/339 (97.6)	90/91 (98.9)	241/248 (97.1)	<0.001
Length of stay, days, median (IQR)	2 (2–5)	2 (1–2)	4 (3–7)	0.250
Follow-up duration, days, median (IQR)	187 (21–428.7)	187 (20–407)	187 (21–431)	0.409
Residual shunt status at recent follow-up, *n* (%)	0 (0)	0 (0)	0 (0)	<0.001
Clinical symptoms during recent follow-up				
Nausea, *n* (%)	4 (1.1)	2 (2.1)	2 (0.8)	
Palpitation, *n* (%)	2 (0.5)	1 (1)	1 (0.4)	
Breathelessness, *n* (%)	10 (2.9)	4 (4.3)	6 (2.4)	
Chest pain, *n* (%)	9 (2.6)	1 (1)	8 (3.2)	

IQR, interquartile range.

**Table 3:** Patient with serious adverse event (SAE).

No	Gender	Age (year)	Weight (kg)	ASD size	Complication	Treatment
1	F	1	9.8	13.6	Wire-induced perforation of the left atrial appendage with cardiac tamponade	Surgical repair
2	F	14	32	28	Ventricular tachycardia with pulse	Cardioversion and amiodarone
3	F	17	41	20	Transient complete AV block (CAVB)	None
4	F	50	46	25	Unstable atrial flutter 1:1	Cardioversion
5	F	9	15	15	Device embolization to right atrium	Device snare-recapture and ASD surgical closure
6	F	9	23	19	Device embolization	Device snare-recapture and redeployment of the ASD device
7	F	22	35	35	Device embolization	Device snare-recapture and ASD surgical closure

ASD, atrial septal defect; CAVB, complete atrioventricular block; F, female.

### 3.3 Outcomes of the zero-fluoroscopy complex ASD closure procedures

Two patients underwent procedures guided solely by TTE. A 14-year-old female experienced ventricular tachycardia (VT) with a pulse during device placement; she was successfully cardioverted and treated with amiodarone, restoring sinus rhythm and stabilizing her hemodynamic status. Two patients experienced transient junctional rhythm, and one patient, who had a history of epilepsy under treatment and global developmental delay, developed a transient high-degree atrioventricular (AV) block during the procedure; however, no significant complaints were reported post-procedure.

Additionally, six pregnant patients underwent exclusively echocardiography-guided ASD closure. A 29-year-old woman with a bidirectional shunt received a fenestrated device. In another case, a 50-year-old woman developed unstable atrial flutter (1:1) following device closure, which was resolved by cardioversion, restoring rhythm first to junctional and then to sinus.

## 4 Discussion

### 4.1 Effectiveness of absolute zero fluoroscopy ASD closure

In existing literature, echocardiographic imaging is commonly cited as an adjunctive imaging modality alongside fluoroscopy for assessing ASD anatomy (9). Ewert et al. (2000) (16) initially reported findings on the viability of interventional ASD device closure without fluoroscopy in a cohort of 26 patients. Subsequently, Schubert et al. (2012) (10) investigated the same approach in 330 patients. Yang et al. (2016) (11) documented outcomes of 114 pediatric cases undergoing transcatheter device closure guided solely by TEE, without fluoroscopy. Despite several prior studies demonstrating comparable success rates to fluoroscopy-guided procedures, this adapted technique has yet to gain widespread acceptance as the standard approach (10–15).

TEE offers intricate assessments of ASD and their surrounding rims, presenting as a feasible, secure, minimally invasive, and straightforward procedure (9). In patient undergoing ASD closure guided solely by TEE, the procedure involves utilizing TEE to direct the passage of a wire from the vena cava to the right atrium. As the wire enters the right atrium, TEE facilitates visualization of its position and aids in guiding it across the ASD, thus establishing a path for the occlusion device (11). Throughout the deployment of the occlusion device, TEE serves as a vigilant monitor, ensuring precise placement of the device, confirming complete release, detecting any residual shunt, and assessing the condition of the atrial valve (11, 17–20). In our practice, once the correct positioning of the occlusion device was confirmed, we fully released the device to evaluate its efficacy.

### 4.2 Technical implications and outcomes of complex ASD cases

Echocardiography enables visual assessment of the impact of the occluder on structures such as the mitral valve, coronary sinus, and pulmonary vein (9, 11). Our study demonstrates the feasibility of performing absolute zero fluoroscopy ASD closure, even in complex cases.

Visible wire, catheter, sheath, and device behavior is essential throughout the procedure, rotating without movement if contact is lost. Device maneuvering requires an understanding of the rim's stability, ideally achieving secure placement within a single attempt. When necessary, the balloon-assisted technique offers a safer alternative, especially for larger ASDs or in cases of device misalignment. In patients with a small LA, the LA approach is preferable, especially in infants with a well-defined rim. LA approach in ASD closure refers to anchoring and deploying the device in the left atrium during the procedure to facilitate defect closure. Exclusive echhocardiography-guidance ASD closure should avoid multiple attempts. In cases where exclusively echocardiography-guidance is insufficient, fluoroscopy should be readily available as a backup.

Failure of the echocardiographer to properly visualize may necessitate adjustments based on procedural progress. In patients with a poor echocardiographic window or during initial experience, conversion may be required. All of our conversion to fluoroscopy occurred during initial learning curve of this procedure.

Seven patients experienced complications, all of which were associated with complex cases. The first case involved a wire perforating the wall of the left atrial appendage; however, the patient survived and underwent successful surgical repair. In the second case, ventricular tachycardia with a pulse occurred during device placement. The patient was successfully cardioverted and treated with amiodarone, resulting in rhythm conversion to sinus and hemodynamic stabilization. The third patient developed transient high-degree atrioventricular block during the procedure. This patient had a history of epilepsy and global developmental delay but reported no significant issues following ASD closure. The fourth case involved unstable atrial flutter with 1:1 atrioventricular conduction after device closure. Cardioversion restored the rhythm, transitioning briefly through junctional rhythm to sinus rhythm. Device dislodgement into the right atrium was noted in the fifth and seventh cases, both of which required surgical retrieval. Finally, in the sixth case, device embolization to the right atrium was successfully managed by snaring and redeployment. Further details on these complications are provided in Table 3. Reports exist of transcatheter ASD closure conducted under TEE guidance without the use of fluoroscopy (6). Qiu et al. (2022) (21) documented the cases of 45 pregnant women who underwent transthoracic echocardiography-guided percutaneous closure of ASD, with all patients experiencing improved right ventricular function post-closure. Furthermore, closure of ASD resulted in an enhancement of quality of life among pregnant women.

### 4.3 Different devices and their features when using zero fluoroscopy

The Amplatzer Septal Occluder (ASO) and ASO-like devices are fully composed of nitinol, making them highly visible on echocardiography. In contrast, newer devices, such as the GORE Cardioform ASD Occluder (WL Gore & Associates, Flagstaff, AZ), have reduced metal density, resulting in a more complex deployment mechanism and less distinct visibility on echocardiography. Published studies on these newer devices commonly report using x-ray guidance, with echocardiography primarily utilized to assess residual shunting rather than to guide device positioning.

The GORE Cardioform ASD Occluder is distinguished by its flexibility and adaptability to various anatomies. Santoro et al. (22) recommend its implantation with both fluoroscopic and echocardiographic guidance to optimize outcomes. The devices used in this study share a similar overall shape and composition, which markedly differ from those of GORE devices. Notably, none of the patients in this cohort were treated with GORE devices. Although our institution did not employ the GORE Cardioform device, we used a comparable flexible device, the Cera™ ASD Occluder (Lifetech, China), under echocardiographic guidance alone, successfully managing intra- and periprocedural phases for secundum ASDs without complications.

### 4.4 Applications of the different complex implantation maneuvers using absolute zero fluoroscopy

Several technical modifications were employed to overcome complex anatomical challenges. These included adjustments in deployment maneuvers. When standard techniques proved ineffective, alternative strategies involved positioning the left disk within the left or right upper lobe pulmonary vein. Further adaptations in implantation techniques included the use of customized or steerable delivery sheaths and the application of balloon-assisted closure methods (23).

Haddad et al. (2023) (24) introduced a novel ASD closure technique called the FAST (Fast Atrial Sheath Traction) method. This technique rapidly unsheathes the device in the left atrium, allowing simultaneous clamping of the ASD from both sides. It was mainly applied in patients with absent aortic rims or when the ASD size-to-body weight ratio exceeded 0.9. At our institution, we occasionally employed a similar technique in complex ASD cases. In these instances, we quickly deployed the device into the pulmonary vein-left atrium, followed by immediate traction to open the posterior (right) disc, achieving a clamping effect. This approach was typically used when conventional methods were unsuccessful.

## 5 Limitations

This single-center retrospective study did not involve randomization. The study design spans a substantial timeframe, allowing for observation over multiple years. Throughout this period, operators' experience levels have likely increased, potentially influencing procedure times as operators become more proficient and efficient. The advancement of interventional skills, the introduction of new device types, and the standardization of procedures among operators may introduce some bias, potentially contributing to fewer technical intraprocedural events and shorter procedure durations. We also only used ASO-like devices. Therefore, this study does not apply to GORE devices.

## 6 Conclusions

Exclusively-echocardiography guided ASD closure is feasible and safe. However, in more complex cases, there is a significantly higher likelihood of conversion to x-ray guidance, along with an increased risk of procedural failure.

## Data Availability

The original contributions presented in the study are included in the article/Supplementary Material, further inquiries can be directed to the corresponding author.
